# Usability of the AAOS Appropriate Use Criteria (AUC) for the surgical management of knee osteoarthritis in clinical practice

**DOI:** 10.1007/s00167-020-05908-7

**Published:** 2020-03-06

**Authors:** Ghalib Oudah Ahmed, Kareem ELSweify, Abdulaziz F. Ahmed

**Affiliations:** Orthopedics Department, Hamad Medical Corporation, Hamad General Hospital, PO Box 3050, Doha, Qatar

**Keywords:** AAOS, AUC, Knee, Osteoarthritis, Osteotomy, Arthroplasty, Appropriateness

## Abstract

**Purpose:**

The Appropriate Use Criteria (AUC) for the surgical treatment of knee osteoarthritis were developed by the American Academy of Orthopedic Surgeons (AAOS) to guide surgeons in selecting the most evidence-based surgical option. This study aimed to assess the usability of the AUC by comparing the actual surgical treatment provided at our institution with that recommended by the AUC.

**Methods:**

A retrospective review of the medical charts and radiographs of all patients who underwent surgery for knee osteoarthritis (OA) at our hospital was performed between January and December 2017. Data including each patient’s age, gender, pain level, mechanical symptoms, range of motion (ROM) and instability, radiographic pattern and severity, limb alignment, and type of surgical interventions received were collected.

The collected data were input into the AUC application to determine the rate of appropriateness of the treatments. Afterwards, the agreement between the actual treatment provided and the AUC recommendation was assessed.

**Results:**

A consecutive series of 100 patients were included. The mean age was 63.1 years, with the majority of the patients aged (73%) between 50 and 69 years. Most of the patients were females (74%), and 61% had left knee OA. The most frequent type of patient was a middle-aged patient with function-limiting pain at short distances, no mechanical symptoms or functional instability with full ROM, severe knee multicompartmental radiographic features, and varus or valgus malalignment. Out of the 100 patients, total knee arthroplasty (TKA) was performed in 85 patients, unicompartmental knee arthroplasty (UKA) was performed in 11 patients, and high tibial osteotomy (HTO) was performed in four patients.

According to the AUC, 90 (90%) cases were treated with an appropriate surgical treatment, whereas 10 (10%) cases were treated with a maybe appropriate treatment. The actual surgical treatment performed at our hospital was in agreement with the AUC recommendation in 100% of the TKA cases, 90.9% of the UKA cases, and 100% of the HTO cases. Thus, the agreement rate with the AUC was 99% in all surgical cases.

**Conclusion:**

This study demonstrated that the AUC for the surgical treatment of knee OA can be applied easily in a clinical setting. Most of the treatments provided at our institution were appropriate and in agreement with the AUC recommendations. Additionally, the AUC had a web-based application that was easy to use and simple for identifying treatment recommendations.

**Level of evidence:**

Retrospective study, level IV.

## Introduction

Knee osteoarthritis (OA) is the fourth leading cause of hospitalization in the United States and accounts for the majority of osteoarthritis cases that require surgical treatment [1–7].

The majority of knee osteoarthritis cases are treated nonsurgically. However, surgical treatment is indicated when patients have significant symptoms that are recalcitrant to nonsurgical treatments [1, 5, 8].

The decision on which surgical option is appropriate depends on several factors, including the patient’s age, symptoms (e.g., pain and knee function), level of physical activity, OA stage, and medical comorbidities and the available evidence. However, radiological evidence of OA alone does not justify surgical intervention [9–11].

In 2015, the American Academy of Orthopedic Surgeons (AAOS) released clinical practice guidelines that are based on the best available evidence to facilitate the decision-making process and improve the quality of care provided in the surgical treatment of knee OA [10, 12].

Subsequently, the AAOS published the Appropriate Use Criteria (AUC) in 2015 for the surgical management of knee osteoarthritis based on relevant expertise and evidence.

The AUC for the surgical management of knee OA assessed eight factors for each patient, and an appropriateness rating was generated for each of the following three interventions: total knee arthroplasty (TKA), unicompartmental knee arthroplasty (UKA), and realignment osteotomy [10, 13, 14].

To the best of our knowledge, no prior studies have investigated the value of the AUC as a clinical tool for the surgical treatment of knee OA in clinical practice.

This study aimed to assess the usability of the AUC by comparing the actual surgical treatment provided at our institution with that recommended by the AUC.

## Materials and methods

This study was conducted at Hamad General Hospital in Qatar. Our institution is accredited by the Joint Commission International and the Accreditation Council for Graduate Medical Education-International, which mandates that all treating physicians properly document all of their patient data. Our institution is the only tertiary care center in Qatar with more than four orthopedic knee surgeons who manage knee OA. Approximately 3000 cases of knee OA are treated annually, and approximately 250 cases of knee reconstructive surgeries are performed annually in our hospital.

A retrospective medical chart review was performed by two authors for 115 consecutive patients who underwent surgical treatment for knee OA between January 2017 and December 2017. The inclusion criteria were adult patients (≥ 18 years) who underwent either TKA, UKA, or high tibial osteotomy (HTO) for primary knee OA. The exclusion criteria were secondary knee OA (e.g., traumatic or inflammatory), a previous surgical intervention (five cases), the presence of neoplasms, neuropathy (one cases), vascular disease, the presence of concomitant ipsilateral hip arthritis or foot deformity (two cases), and incomplete documentation (seven cases).

The AUC application requires eight patient parameters to generate appropriateness ratings for three surgical treatment options (TKA, UKA, and realignment osteotomy) for knee osteoarthritis. Each treatment is rated as appropriate, may be appropriate, or rarely appropriate according to the AUC application.

Thus, the eight parameters according to the criteria of the AUC were retrieved by two authors for 100 consecutive patients included in this study. The patient’s age, gender, pain level, knee range of motion, knee instability, mechanical symptoms, number of affected osteoarthritic compartments, radiographic severity of knee OA using the Kellgren−Lawrence (KL) grading system, radiographic limb alignment, and surgical treatment provided were collected.

The mean age was 63.1 years, the majority of patients (73%) were aged 50−69 years, 74% of the participants identified as female, and 61% had left-sided knee osteoarthritis. Table 1 summarizes the patients’ characteristics.

**Table 1:** Patient’s characteristics

Mean age (range)	63.1 years (23–91)
Young (< 49 years)	3%
Middle-aged (50–69 years)	73%
Elderly (> 70 years)	24%
Sex	%
Male/female	26%/74%
Side of OA	%
Right/left	39%/61%
Pattern of arthritic involvement	%
More than one compartment	85%
One compartment	15%
KL radiographic grading of knee OA^a^	%
Severe	85%
Mild-to-moderate	15%
Limb alignment	%
Normal	26%
Varus	69%
Valgus	5%
Function limiting pain	%
Pain at rest	36%
At short distance (walking less than ¼ mile)	44%
At moderate-to-long distances (walking greater than 1/4 mile)	20%
Range of motion (extension/flexion)	%
Full range of extension/flexion	53%
Flexion contracture > 5° and/or flexion < < 110°	41%
Flexion contracture > 10° and/or flexion < < 90°	6%
Functional instability^b^	%
No functional instability	100%
Mechanical symptoms^c^	%
No mechanical symptoms	100%
Type of surgical intervention	N (%)
TKA	85 (85%)
UKA	11 (11%)
HTO	4 (4%)

*OA* osteoarthritis

^a^Severity was assessed using joint space narrowing, osteophyte formation using the Kellgren−Lawrence (K−L) scale from a weight bearing AP, and lateral standing radiograph. Grade 1 and 2 considered as mild OA, grade 3 as moderate OA, and grade 4 as severe OA

^b^Functional instability was defined as sudden loss of postural support across the knee at the time of weight bearing such as feeling of buckling, lack of confidence, and/or giving way of the knee that affect the daily life activity.

^c^Mechanical symptoms were defined as a resistance to knee motion such as catching and/or locking of the knee that caused by something being trapped inside the knee joint.

To assess the usability of the AUC for the surgical management of knee OA, first, the parameters of each patient were input into the AUC to generate the appropriateness rating of the provided treatment for each patient. Afterwards, the agreement between the actual surgical treatment provided at our institution and the AUC recommendations was assessed.

The Medical Research Center review board (Hamad Medical Corporation, reference number MRC-01-18-1320) approved the study prior to the initiation of this study.

### Statistical analysis

The statistical software (IBM SPSS version 24; SPSS Inc., Chicago, IL, USA) was used for data analysis.

Descriptive statistics such as the mean, range and percentage were used to summarize the patients’ demographics and treatment options.

The appropriateness rating for each treatment whether it was appropriate or may be appropriate was described with percentages. The agreement of the treatments implemented at our institution with the AUC recommendations was expressed as a proportion.

The accuracy of the data collection was evaluated by comparing the data collection process performed by two authors. Afterwards, a Pearson’s correlation coefficient was conducted, and an intraclass correlation coefficient (ICC) > 0.75 was considered to indicate excellent agreement.

No sample size calculations were performed before conducting this study, because all patients who met the inclusion criteria were included. A post hoc power analysis revealed a power of greater than 80%, which indicated that the sample size was adequate for analysis.

## Results

Out of the 100 patients included in this study, TKA was performed in 85 (85%) patients, UKA was performed in 11 (11%) patients, and HTO was performed in 4 (4%) patients. Surgeries were performed by fellowship and non-fellowship-trained adult reconstruction surgeons.

The most frequent type of patient was a middle-aged patient with function-limiting pain at short distances (limiting activity to two city blocks or the equivalent to walking the length of less than ¼ mile), no mechanical symptoms, full range of extension/flexion, no functional instability, more than one compartment, severe knee OA (KL grade IV), and varus or valgus malalignment (Table 1).

Of the included patients, 90 (90%) were treated with an appropriate surgical treatment, whereas 10 (10%) were treated with a maybe appropriate treatment, as determined by the AUC application. No cases were treated with rarely appropriate treatments.

When each treatment was assessed individually, TKA was rated as appropriate in 97.6% (*N* = 83) of the cases and may be appropriate in 2.4% (*N* = 2) of the cases.

The UKA was rated as appropriate in 54.5% (*N* = 6) of the cases and may be appropriate in 45.5% (*N* = 5) of the cases, whereas HTO was rated as appropriate in 25% (*N* = 1) of the cases and may be appropriate in 75% (*N* = 3) of the cases (Table 2).

**Table 2:** Summary of the surgical treatments’ appropriateness and agreement with the *AUC* recommendations

Surgical treatment	*N*	Appropriateness rating		Agreement with the AUC Recommendations
		Appropriate	May be appropriate	
Overall	100	90%	10%	99%
TKA	85	83 (97.6%)	2 (2.4%)	85 (100%)
UKA	11	6 (54.5%)	5 (45.5%)	10 (90.9%)
HTO	4	1 (25%)	3 (75%)	4 (100%)

*TKA *total knee arthroplasty, *UKA* unicompartmental knee arthroplasty, *HTO *high tibial osteotomy

The actual surgical management was in agreement with the AUC recommendation in 99 (99%) of treatment. By grouping the surgical interventions, the agreement with the AUC recommendations was found in 85 (100%) cases of TKA, 10 (90.9%) cases of UKA, and 4 (100%) cases of HTO (Table 2).

Regarding the accuracy of the data collection, the ICC was 1.00, indicating excellent agreement between the two authors who performed the data collection.

## Discussion

The most important finding of the present study was that the application of the AUC made selecting an appropriate surgical treatment for each patient relatively simple and feasible. All AUC-recommended surgical treatment options were performed in our patients, with a predominance of TKA.

The treatment provided at our hospital was found to be appropriate and in agreement with the AUC recommendations in the majority of patients, although none of the orthopedic surgeons at our institute used the AUC preoperatively. This finding demonstrated the consensus regarding surgical treatment for knee OA at our institute with evidence-based indications.

For patients who underwent TKA, this treatment was appropriate in the overwhelming majority of cases (97.6%), whereas patients who underwent UKA and HTO had a low appropriate rate. This result and our patients’ demographics indicate that the TKA is the standard surgical option for older individuals with advanced knee OA [15]. However, the agreement with the AUC recommendation for all cases was 99% (*N* = 99) despite that ten cases out of these agreed upon cases were managed with a, maybe, appropriate rating. This is because the AUC recommendations for these cases were, maybe, appropriate at maximum with no more appropriate treatments advised by the AUC.

When examining the UKA cases, we found that this procedure was considered appropriate for 54.5% of the cases, because the AUC does not recommend UKA for patients with valgus or varus malalignment. This finding might reflect a shortcoming in the AAOS-published AUC, as it does not quantify the degree of malalignment. Instead, it accounts for malalignment as a dichotomous variable. It is noteworthy that the standard prerequisites to perform a UKA include malalignment that is less than 10° of valgus or > 5° of varus. Furthermore, a recent meta-analysis found that UKA resulted in superior outcomes at 6 and 12 months compared to TKA in appropriately selected patients, although the population had variable degrees of varus and valgus knee malalignment [16]. Hence, the vagueness in the definitions of malalignment in the AAOS-published AUC should be clarified.

Similarly, HTO was rated maybe appropriate in most of cases due to the patient’s older age or the presence of any valgus or varus malalignment. The treatment performed was considered maybe appropriate for more UKA and HTO patients than TKA patients due to the presence of varus or valgus malalignment combined with mild-to-moderate knee OA. The absence of a defined range of malalignment in the AUC may have led to the treatment being considered maybe appropriate instead of appropriate.

The use of evidence-based clinical tools, such as the AUC, in this subgroup of patients provided an opportunity to improve the quality of clinical practice by guiding orthopedic surgeons in selecting an appropriate treatment. Hence, the variation in the surgical treatments selected for patients with knee OA should be decreased, and patient outcomes should be improved.

In this study, the use of AUC was feasible, because it was easily accessible through a web-based application and enabled surgeons to evaluate the appropriateness of their treatment.

The tool might also be advantageous for surgeons with a small amount of experience, as it can recommend a treatment that is consistent with the best available evidence, especially for cases that might be eligible for UKA and HTO.

Several drawbacks of the AUC for the surgical treatment of knee OA were identified;

Limb alignment (i.e., varus or valgus) is not clearly defined, and it may affect the appropriateness of treatments substantially, although the literature has reported that there are acceptable degrees of both varus and valgus malalignment that do not affect the surgical outcome [16]. Another shortcoming is that patient weight was not accounted for in the AUC criteria, which might influence the surgical decision. For example, morbid obesity might lead to deleterious results in patients who undergo HTO or UKA for knee OA.

This study has several limitations. Due to the retrospective nature of the study, no functional outcomes were collected. Additionally, incomplete documentation of medical charts can hinder the application of the AUC. Patients with incomplete documentation were excluded, because the AUC relies on eight patient parameters to be accurately documented; however, this patient selection process reduced the overall sample size. Subsequently, a small number of UKA and HTO cases were included in the study, which might have affected the appropriateness of these cases. An additional source of bias may be the variability in the interpretation of knee OA severity from the radiographs.

## Conclusion

This study demonstrated that the AUC for surgical treatment of knee OA is easy to use in a clinical setting. Most of the treatments provided at our institution were appropriate and in agreement with the AUC recommendations. Additionally, the AUC had a web-based application that was easy to use and simple for identifying treatment recommendations.
